# T-cell tumour exclusion and immunotherapy resistance: a role for CAF targeting

**DOI:** 10.1038/s41416-020-1020-6

**Published:** 2020-08-24

**Authors:** Christopher J. Hanley, Gareth J. Thomas

**Affiliations:** 1 School of Cancer Sciences, University of Southampton, Southampton, UK; 2 Cancer Research UK and NIHR Southampton Experimental Cancer Medicine Centre, Southampton, UK

**Keywords:** Cancer microenvironment, Tumour immunology

## Abstract

Recent studies have highlighted a major role for cancer-associated fibroblasts (CAFs) in promoting immunotherapy resistance by excluding T cells from tumours. Recently, we showed that CAFs can be effectively targeted by inhibiting the enzyme NOX4; this ‘normalises’ CAFs and overcomes immunotherapy resistance. Here we discuss our study and other strategies for CAF targeting.

## Main

The effectiveness of checkpoint immunotherapy using blocking antibodies against CTLA-4 and PD1/PD-L1 is established, but only a minority of patients with certain cancers respond. The success of these inhibitors is predicated upon reactivating a pre-existing antitumour immune response. Morphologically, this manifests as a dense infiltrate of T cells within the tumour, and such ‘inflamed’ tumours typically respond well to checkpoint inhibitors. However, non-inflamed tumours are often resistant, and can be separated into two morphologically distinct groups: ‘desert’ tumours, with very few immune cells, and ‘excluded’ tumours, where T cells are present, but restricted to the tumour periphery. Recent studies have suggested a role for cancer-associated fibroblasts (CAFs) in mediating this exclusion.^1–3^ Targeting CAFs could therefore benefit a significant group of cancer patients that would normally be resistant to immunotherapy.

CAFs are associated with poor prognosis in many tumour types.^4^ The term ‘CAF’ mostly refers to a cell with an ‘activated’ myofibroblastic phenotype. These cells are αSMA-positive, contractile and secrete extracellular matrix (ECM), analogous to myofibroblasts found in healing wounds and fibrotic lesions. Recently, we developed CAF-rich murine tumour models to investigate the effect of CAF on the immune microenvironment and immunotherapy response.^3^ This showed that CD8 T cells fail to infiltrate CAF-rich tumours, instead accumulating at the tumour margin, and upregulating expression of CTLA-4. This exclusion results in resistance to multiple immunotherapies, including therapeutic vaccination and αPD1. We had shown previously that the ROS-producing enzyme NADPH oxidase-4 (NOX4) regulates myofibroblastic CAF differentiation in multiple cancers,^4^ and that NOX4 inhibition can revert myofibroblastic CAFs to a quiescent, fibroblast-like phenotype.^3,4^ Therefore, we examined whether targeting this pathway, using a small-molecule NOX4/1 inhibitor (GKT137831 [Setanaxib], Genyotex), could overcome CAF-mediated immunotherapy resistance. This drug effectively reversed CAF differentiation in vivo, significantly promoted infiltration of CD8 T cells into tumours and potentiated the response to immunotherapy.^3^

To date, attempts to therapeutically target CAFs have been unsuccessful. To improve the chances of translating preclinical findings into patient benefit, improved model systems are required that more accurately recapitulate the human tumour microenvironment. We found that common murine tumour models often lack the stromal reaction observed in human tumours, and therefore developed CAF-rich models to more accurately model this feature.^3^ The challenge of targeting CAFs is further compounded by their heterogeneity. Recently, single-cell RNA-seq has been used to characterise multiple subpopulations, including identification of inflammatory and myofibroblastic CAFs across cancer types (pancreatic, lung and breast cancers^5–7^). These studies have elucidated markers and immune-phenotyping strategies for identifying these subpopulations, which will enable increasingly precise mechanistic analysis of CAFs in future studies.

**Fig. 1:**
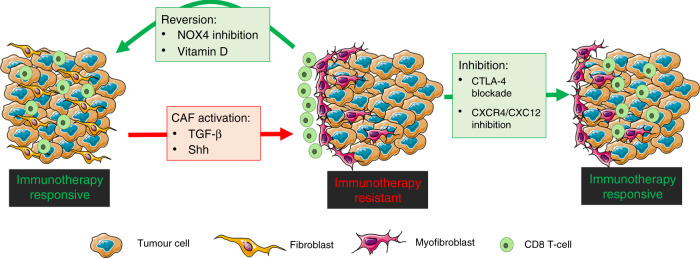
Schematic showing possible strategies for targeting CAFs and overcoming CAF-mediated T-cell exclusion from tumours. Strategies include inhibiting CAF differentiation (TGF-β & Shh inhibitors) reverting CAF to a more ‘normal’ phenoype (NOX4 inhibitors, vitamin D agonists) or inhibition CAF function (CXCR4/CXCL12 inhibitors).

Myofibroblastic CAFs have been linked to immunotherapy resistance in multiple studies.^2,6,8^ Due to fibroblast plasticity, an attractive therapeutic strategy is to revert myofibroblastic CAF to a more ‘normal’ or even tumour-suppressive phenotype (Fig. 1). In order to develop strategies for this, we must identify the molecular mechanisms that maintain (rather than initiate) CAF activation. TGF-β signalling is central to myofibroblast differentiation, and has therefore gathered much interest as a potential method for targeting CAFs. Mariathasan and colleagues showed that TGF-β1-neutralising mAbs can promote lymphocyte infiltration into tumours and improve response to αPD-L1 immunotherapy.^2^ In our CAF-rich models, we similarly found that TGF-β1 inhibition increases intratumoural CD8 T-cell density. However, this was not associated with reduced CAF levels or reduced CD8 T cells at the tumour margin, indicating a CAF- and exclusion-independent mechanism.^3^ In support of this, in vitro analysis showed that TGF-β1 inhibition prevents CAF activation, but does not reverse the established CAF phenotype.^3^ NOX4 is known to act downstream of TGF-β1, and its inhibition was sufficient to reverse CAF differentiaion and ‘normalise’ the cell, suggesting that investigating downstream mediators of TGF-β1 signalling could lead to further identification of novel therapeutic targets. An alternative strategy is to activate pathways that regulate ‘normal’ precursor cell phenocytes. Vitamin D agonists have been identified as a potential method to achieve this in pancreatic cancer,^9^ and are currently under investigation in an ongoing clinical trial (NCT03520790). Hedgehog signalling is another pathway that has been extensively explored in pancreatic cancer. However, despite promising preclinical data, hedgehog inhibitors failed in a clinical trial (NCT01130142), and deletion of tumour-cell-derived Shh was found to result in more aggressive cancer growth.^10^

Identifying targetable mechanisms by which CAFs modulate tumour-immune evasion is also a potential therapeutic strategy (Fig. 1). CXCL12 expression by CAFs has been shown to mediate immunotherapy resistance and T-cell exclusion. CXCR4 (CXCL12 receptor) inhibition using a clinically approved inhibitor (AMD3100) has been shown to increase intratumoural T-cell accumulation and response to checkpoint inhibition in models of pancreatic and breast cancer.^1,11^ We found that CAFs induce CTLA-4 upregulation in CD8 T cells, and that blocking CTLA-4 with a non-depleting antibody (i.e. not decreasing T regs) overcomes CAF-mediated T-cell exclusion, without affecting CAF levels.^3^ CTLA-4 has been shown to modulate T-cell adhesion and migration, and therefore blocking CTLA-4-mediated adhesion may improve T-cell trafficking into excluded CAF-rich tumours. Further work in this area is required.

In summary, CAF-rich tumours are clinically aggressive and respond poorly to immunotherapy, at least in part because of T-cell exclusion. Overcoming CAF-mediated immune evasion is a major challenge as the success of most immunotherapies is dependent on CD8^+^ T-cell-infiltrating tumours. We have shown that NOX4 inhibition using GKT137831 can revert myofibroblastic CAFs to a ‘normalised’ phenotype. A significant proportion of solid cancers are CAF-rich, and combining NOX4 inhibition with immunotherapy could improve clinical outcome in these tumours. Notably, GKT137831 is an oral drug with an excellent safety profile developed as an anti-fibrotic (NCT03226067 and NCT02010242). Its use for cancer therapy has not been considered but may be considerable.

## Data Availability

Not applicable.
